# Case Report: extensive leptomeningeal metastasis from gastric cancer presenting with hearing impairment

**DOI:** 10.3389/fonc.2026.1888134

**Published:** 2026-07-08

**Authors:** Yan-cheng Lin, Xiang Li, Xi-chang Zhan, Wan-hui Dong

**Affiliations:** Department of Medical Oncology, Lu’an Hospital of Traditional Chinese Medicine Affiliated to Anhui University of Chinese Medicine, Lu’an, China

**Keywords:** case report, contrast-enhanced MRI, extensive, gastric cancer, hearing impairment, HER2, leptomeningeal metastasis

## Abstract

Leptomeningeal metastasis (LM) from gastric cancer is a rare but devastating manifestation of advanced disease. Early diagnosis can be difficult when neurological symptoms are atypical and cerebrospinal fluid (CSF) cytology is negative. We report a 71-year-old man with gastric cardia adenocarcinoma who initially developed hearing impairment, dizziness and gait instability approximately 11 months after radical total gastrectomy, followed by rapidly progressive bilateral hearing loss at presentation. Neurological examination initially showed no meningeal signs or pathological reflexes. Contrast-enhanced brain magnetic resonance imaging (MRI) demonstrated extensive, multifocal linear and patchy leptomeningeal enhancement over the bilateral cerebral hemispheres and cerebellar surfaces. CSF analysis showed marked hypoglycorrhachia, elevated protein, and mild mononuclear pleocytosis, whereas cytology did not identify malignant cells. After alternative infectious and immune-related diagnoses were considered clinically less likely, the findings supported a diagnosis of probable type IIA LM according to the EANO-ESMO criteria. Immunohistochemistry of the primary gastric tumor showed CK7 positivity, diffuse CAM5.2 positivity, partial CDX-2 nuclear positivity, HER2 immunohistochemistry (IHC) 2+ membranous staining, mutant-pattern p53 expression, and a Ki-67 proliferation index of approximately 90%. The disease progressed rapidly to impaired consciousness, and the family chose best supportive care after a multidisciplinary team (MDT) discussion. This case highlights that leptomeningeal metastasis from gastric cancer may initially present with hearing impairment; integrated assessment of MRI findings, CSF biochemistry, clinical course, and careful consideration of alternative diagnoses is essential.

## 1 Introduction

Gastric cancer is the fifth most commonly diagnosed malignancy worldwide and the fourth leading cause of cancer-related mortality (1). Although central nervous system involvement is uncommon in gastric cancer, improvements in systemic therapy and prolonged survival have increased clinical attention to brain and meningeal metastases. Intracranial involvement from gastric carcinoma includes parenchymal brain metastasis and leptomeningeal metastasis (LM), also referred to as leptomeningeal carcinomatosis, in which tumor cells infiltrate the pia mater, arachnoid, and subarachnoid space (2–4). Brain metastases from gastric cancer occur in approximately 0.5%-2.29% of patients, whereas LM accounts for only 0.06%-0.19% of gastric cancer cases (5, 6). The prognosis of LM is poor, with reported median survival of only 4–6 weeks without treatment and only 2–3 months even with active therapy in historical series (7).

Diagnosis remains challenging because clinical manifestations may be nonspecific and cerebrospinal fluid (CSF) cytology has limited sensitivity (8). The EANO-ESMO guideline recognizes a probable LM category when compatible clinical and neuroimaging findings are present and alternative diagnoses have been reasonably excluded, even in the absence of positive CSF cytology (9). Here, we describe an older patient with gastric cancer who developed hearing impairment, dizziness and gait instability, with hearing impairment progressing to bilateral hearing loss at presentation, had negative CSF cytology, and showed extensive leptomeningeal enhancement on contrast-enhanced magnetic resonance imaging (MRI).

## 2 Case presentation

### 2.1 Patient information and clinical presentation

A 71-year-old man was admitted on May 14, 2025, because of severe anemia. Admission laboratory tests showed severe microcytic hypochromic iron-deficiency anemia (hemoglobin 48 g/L, red blood cells 2.87 × 10^12^/L, hematocrit 18.10%, mean corpuscular volume 63.0 fL, mean corpuscular hemoglobin 16.8 pg, and mean corpuscular hemoglobin concentration 267 g/L). Hematinic work-up showed low ferritin (4.60 ng/mL), low serum iron (1.23 μmol/L), unsaturated iron-binding capacity of 67.33 μmol/L, total iron-binding capacity of 68.56 μmol/L, vitamin B12 of 730 pg/mL, and folate of 9.12 ng/mL. Gastroscopy and biopsy confirmed moderately to poorly differentiated ulcerative adenocarcinoma of the gastric cardia, measuring approximately 6 cm × 5 cm × 2 cm. Pathological staging after surgery was pT3N1M0. Immunohistochemistry of the gastric tumor in the initial gastrectomy specimen showed cytoplasmic positivity for CK7, diffuse cytoplasmic positivity for CAM5.2, partial nuclear positivity for CDX-2, HER2 immunohistochemistry (IHC) 2+ membranous staining, nuclear p53 positivity consistent with a mutant expression pattern, and a Ki-67 proliferation index of approximately 90% (**Figure 1**). The patient had no family history of cancer. On May 29, 2025, he underwent radical total gastrectomy. The postoperative course was complicated by septic shock, from which he recovered after comprehensive treatment in the intensive care unit. He subsequently received four cycles of oxaliplatin plus capecitabine.

**Figure 1:**
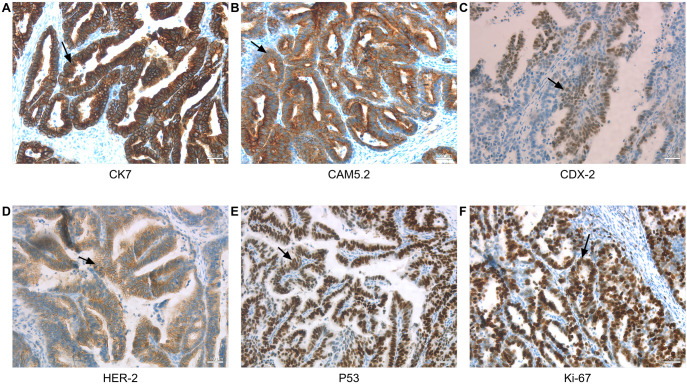
Immunohistochemistry of the gastric tumor. **(A)** CK7: tumor cells show cytoplasmic positivity. **(B)** CAM5.2: tumor cells show diffuse cytoplasmic positivity. **(C)** CDX-2: tumor cells show partial nuclear positivity. **(D)** HER2: tumor cells show IHC 2+ membranous staining. **(E)** p53: tumor cells show nuclear positivity, consistent with mutant-pattern expression. **(F)** Ki-67: the tumor cell proliferation index is approximately 90%, with nuclear positivity. Scale bar: 50 μm, applicable to all panels.

On April 16, 2026 (postoperative day 322), the patient was admitted. Four days before admission, the patient developed sudden hearing impairment, dizziness and unsteady gait, with the hearing impairment progressing to marked bilateral hearing loss by the time of hospital presentation. On neurological examination at admission, he was conscious. No additional neurological abnormalities were documented beyond the presenting hearing impairment, dizziness, and unsteady gait. Cardiopulmonary and abdominal examinations were unremarkable. Meningeal signs were negative, and pathological reflexes were not elicited. Acute cerebral infarction was initially suspected. Because of the prominent hearing loss, an otolaryngology consultation was requested. Fiberoptic otoscopy showed intact tympanic membranes bilaterally and did not suggest a conductive cause of hearing loss, raising concern for an intracranial process. Serial serum carcinoembryonic antigen (CEA) levels increased during follow-up (**Table 1**; **Figure 2**). Although serial CEA levels increased during the four cycles of postoperative chemotherapy, no imaging examination was performed during this chemotherapy period.

**Table 1:** Clinical timeline of the patient.

Clinical category	Time after surgery (days)	Date	Event or intervention	Relevant data
Surgery	0	May 29, 2025	Radical total gastrectomy	N/A
Chemotherapy	75	Aug. 12, 2025	Cycle 1 oxaliplatin plus capecitabine	CEA: 36.32 ng/mL
Chemotherapy	102	Sep. 8, 2025	Cycle 2 oxaliplatin plus capecitabine	CEA: 35.75 ng/mL
Chemotherapy	138	Oct. 14, 2025	Cycle 3 oxaliplatin plus capecitabine	CEA: 41.61 ng/mL
Chemotherapy	181	Nov. 26, 2025	Cycle 4 oxaliplatin plus capecitabine	CEA: 64.33 ng/mL
Neurological presentation	322	Apr. 16, 2026	Admission after hearing impairment, dizziness and gait instability, with progression to marked bilateral hearing loss	N/A
Diagnostic assessment	327	Apr. 21, 2026	Contrast-enhanced MRI and CSF findings supported probable type IIA LM from gastric cancer	N/A
Tumor marker follow-up	341	May 5, 2026	N/A	CEA: 94.91 ng/mL

**Figure 2:**
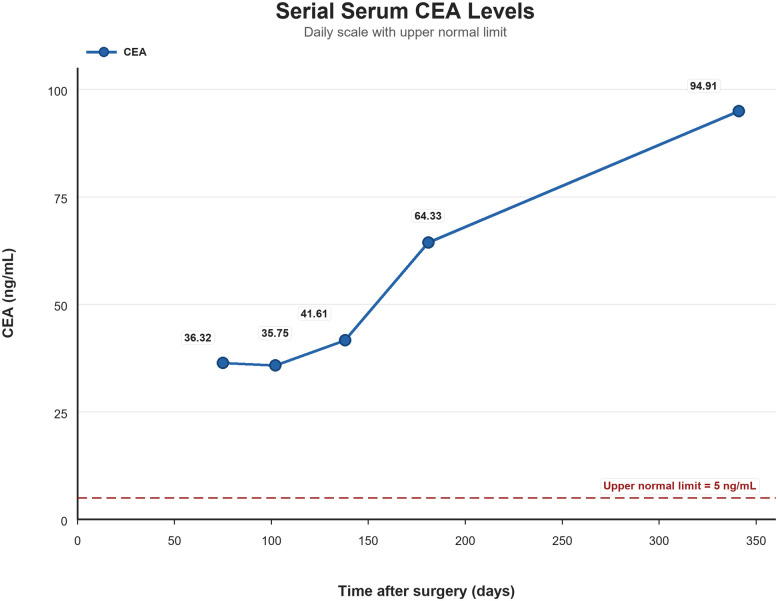
Serial postoperative serum CEA levels. The x-axis indicates time after surgery in days, and the dashed line indicates the upper normal limit of 5 ng/mL. Calendar dates are provided in **Table 1**.

### 2.2 Diagnostic assessment

On admission day 6, lumbar puncture showed an opening pressure of 100 mmH_2_O. CSF analysis showed markedly decreased glucose (0.37 mmol/L), elevated protein (1547.00 mg/L), normal adenosine deaminase (2.10 U/L), a positive Pandy test (1+), white blood cells of 28.00 × 10^6^/L with mononuclear predominance (67.80%), and red blood cells of 2000.00 × 10^6^/L. Non-contrast computed tomography (CT) scans of the head, chest, abdomen, and pelvis showed no mass-forming lesions suggestive of tumor recurrence or systemic metastasis. Brain MRI initially showed an abnormal signal focus in the left frontal lobe. Contrast-enhanced T1-weighted MRI subsequently demonstrated diffuse leptomeningeal thickening and enhancement over the bilateral cerebral hemispheres and cerebellar surfaces, predominantly presenting as multifocal linear and patchy enhancement along the cerebral sulci, Sylvian fissures, and cerebellar surfaces. Bilateral enhancement of the vestibulocochlear nerves (CN VIII) is seen in the cerebellopontine angle cisterns (**Figure 3**). CSF cytology did not identify malignant cells. Based on the compatible clinical features, negative CSF cytology, linear leptomeningeal enhancement on contrast-enhanced MRI, CSF abnormalities, and infectious and immune-related alternative diagnoses considered clinically less likely, the findings supported a diagnosis of probable type IIA LM according to the EANO-ESMO criteria (9).

**Figure 3:**
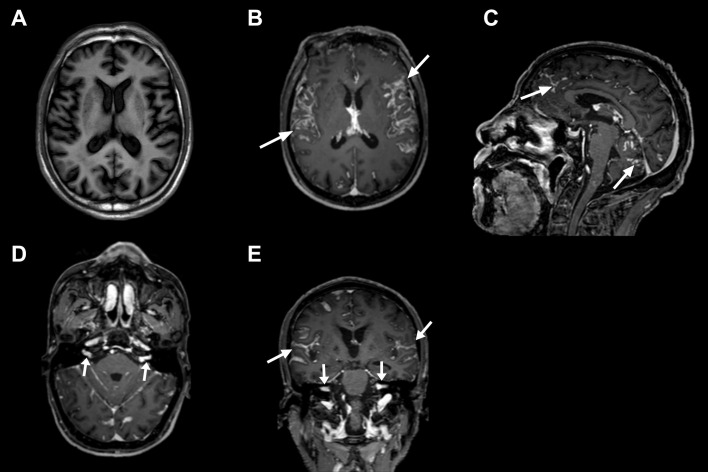
Brain MRI findings. **(A)** Corresponding pre-contrast axial T1-weighted image at the level of **(B)**. **(B, C, E)** Post-contrast T1-weighted images show multifocal linear and patchy leptomeningeal enhancement along the bilateral cerebral sulci, Sylvian fissures, and cerebellar surfaces. **(D, E)** Bilateral enhancement of the vestibulocochlear nerves (CN VIII) is seen in the cerebellopontine angle cisterns. White arrows indicate representative abnormal enhancement.

### 2.3 Therapeutic intervention, follow-up, and outcomes

After probable LM was identified, the attending team recommended further molecular testing, including fluorescence *in situ* hybridization (FISH) for HER2 status and broader genetic testing, to guide possible subsequent therapy. Oxaliplatin plus capecitabine was administered. However, the patient deteriorated rapidly. On admission day 14, he developed impaired consciousness, delirium, sluggish pupillary light reflexes, posterior tongue displacement, and open-mouth breathing. Because of the rapid neurological decline and poor expected benefit of further antitumor treatment, the family declined additional chemotherapy, intrathecal therapy, radiotherapy, and molecular testing after a multidisciplinary team (MDT) discussion. Best supportive care was provided. On admission day 24, his Glasgow Coma Scale (GCS) score was 9 (E4V1M4). On admission day 26, at the family’s request, the patient was discharged home for comfort-focused care. No subsequent follow-up information was available, and survival after discharge could not be ascertained.

## 3 Discussion

### 3.1 Rarity and routes of dissemination

LM from gastric cancer is an exceptionally rare complication, with an estimated incidence of 0.06%-0.19% among patients with gastric cancer (6). It usually occurs late in the disease course. In the present case, the interval between gastric cancer diagnosis and LM was approximately 11 months, consistent with reported median intervals of 10.9-13.1 months (6). Potential routes of meningeal dissemination include hematogenous spread, direct invasion, perineural or lymphatic spread, and rupture of parenchymal brain metastases into the subarachnoid space (10). The postoperative pathological finding of vascular tumor emboli in this patient supports hematogenous dissemination as a plausible route.

### 3.2 Clinical presentation and hearing symptoms

The clinical presentation of LM from gastric cancer is heterogeneous. Reported symptoms include headache, nausea or vomiting, dizziness, cranial nerve palsy, and altered consciousness (6). Hearing loss is uncommon, with one multicenter series of gastric cancer-associated leptomeningeal carcinomatosis reporting a frequency of only 3.7% (11). Sudden hearing loss as an early symptom is particularly rare and may be mistaken for sudden sensorineural hearing loss or drug-related ototoxicity (12). In the present case, bilateral enhancement of the vestibulocochlear nerves (CN VIII) is seen in the cerebellopontine angle cisterns, concordant with the patient’s initial hearing loss and vertigo, a pattern that has been described in LM from gastric cancer (12–14).

### 3.3 Diagnostic integration in cytology-negative disease

CSF cytology remains a diagnostic standard for LM, but its sensitivity is limited. The positivity rate after the first lumbar puncture is approximately 44%-67%, and repeated sampling can increase sensitivity to 84%-91% (8, 15, 16). This limitation is important in cytology-negative cases with strong radiographic and biochemical evidence. In the present patient, CSF protein was markedly elevated and glucose was substantially decreased, supporting meningeal disease despite negative cytology. Tuberculous, bacterial, cryptococcal, and immune-related meningitis were considered. No additional CSF microbiological testing was performed, so infectious meningitis could not be definitively excluded microbiologically. Normal CSF adenosine deaminase, absence of fever or active tuberculosis evidence, mild mononuclear pleocytosis rather than typical purulent CSF, normal opening pressure, absence of imaging features suggestive of cryptococcal meningitis, and no exposure to immune checkpoint inhibitors or related drugs made these alternatives less likely (17–20). Recent meningitis guidance also indicates that older patients may have nonspecific presentations and that classic meningeal signs may be absent or difficult to identify; therefore, negative meningeal signs should not reduce vigilance for meningeal involvement (21, 22). In frail older patients, systemic illness and impaired cooperation may obscure signs.

### 3.4 MRI pattern and disease extent

The MRI pattern in this case was extensive. A recent retrospective series reported that 51.9% of MRI-positive patients with LM from gastric cancer showed diffuse leptomeningeal enhancement (6). Our patient’s MRI showed bilateral, multifocal, and widespread involvement of the cerebral hemispheres and cerebellar surfaces, with linear and patchy leptomeningeal enhancement. This broad distribution indicates a high radiographic disease burden and appears relatively uncommon among published case reports of LM from gastric cancer (4).

### 3.5 HER2 status and molecular limitations

The HER2 status of this case also requires cautious interpretation. The primary gastric tumor showed HER2 immunohistochemistry (IHC) 2+ membranous staining, but confirmatory ISH/FISH testing was not performed. Therefore, the tumor should be regarded as HER2-equivocal rather than definitively HER2-positive. HER2 pathway activation can promote tumor cell survival and metastatic potential in gastric cancer (23), but current evidence does not establish HER2-equivocal status as a proven risk factor for gastric cancer-associated LM. The inability to obtain confirmatory HER2 testing and broader molecular profiling because of rapid clinical deterioration is a limitation of this report.

### 3.6 Treatment considerations and prognosis

Treatment for LM from gastric cancer remains difficult, and no uniform standard based on high-level evidence exists. The EANO-ESMO guideline and the Chinese expert consensus recommend individualized management according to performance status, systemic tumor burden, molecular features, neuroimaging pattern, CSF flow, and neurological symptoms. Options may include systemic therapy, intrathecal or CSF-directed therapy, focal or whole-brain/craniospinal radiotherapy, CSF diversion, and best supportive care (10, 24). For LM from gastric cancer, intrathecal methotrexate or cytarabine may be considered in selected patients, but the level of evidence is low (24). Retrospective series and case reports suggest that intrathecal chemotherapy combined with other treatments may offer benefit in selected patients whose performance status and CSF circulation permit treatment, but this evidence remains limited (6, 11, 25). In a recent single-center series of LM from gastric cancer, median overall survival was only 6.0 weeks, and age ≥60 years and ECOG performance status >2 were independent poor prognostic factors (6). The present patient’s advanced age, rapid progression from hearing impairment, dizziness and gait instability to impaired consciousness, and inability to receive definitive LM-directed treatment were all consistent with poor prognosis.

## 4 Conclusion

In this case, the patient developed rare and extensive LM from gastric cancer, with extremely rapid disease progression but an atypical initial clinical presentation. In older and frail patients, negative meningeal signs may be misleading. When CSF cytology does not show definite malignant cells, contrast-enhanced MRI, CSF biochemical abnormalities, and careful differential diagnosis are essential for timely recognition. Although systemic therapy, intrathecal therapy, and radiotherapy may be considered in selected patients, the prognosis remains poor, and treatment decisions should be individualized through multidisciplinary team (MDT) discussion.

## Patient perspective

Because the patient lacked decision-making capacity after rapid neurological deterioration, the perspective was provided by his family. After the MDT objectively reviewed the imaging findings and explained the irreversible terminal nature of the disease, the family understood, in the context of the patient’s clinical course, that further antitumor intervention was unlikely to provide meaningful survival benefit. To avoid futile suffering and preserve comfort and dignity, the family ultimately chose best supportive care.

## Data Availability

The original contributions presented in the study are included in the article/**Supplementary Material**. Further inquiries can be directed to the corresponding author.
