# Supplementary material for: Case Report: extensive leptomeningeal metastasis from gastric cancer presenting with hearing impairment
Source: Front Oncol. 2026 Jul 8;16:1888134. doi: 10.3389/fonc.2026.1888134 (PMC13388219; doi:10.3389/fonc.2026.1888134)
Supplement: Supplementary file 1 [file Table1.docx]

**CARE Checklist**

| **Topic** | **Item** | **Checklist item description** | **Reported on Line** |
| --- | --- | --- | --- |
| Title | **1** | The words "case report" should be in the title along with the area of focus. | p.1, Title |
| Key Words | **2** | 2 to 5 key words that identify diagnoses or interventions in this case report, including "case report". | p.1, Keywords |
| Abstract | **3a** | Introduction - What is unique about this case? What does it add to the medical literature? | p.1, Abstract |
| Abstract | **3b** | The patient's main concerns and important clinical findings. | p.1, Abstract |
| Abstract | **3c** | The main diagnoses, therapeutic interventions, and outcomes. | p.1, Abstract |
| Abstract | **3d** | Conclusion - What are one or more "take-away" lessons? | p.1, Abstract |
| Introduction | **4** | Briefly summarize why this case is unique and may include medical literature references. | p.2, Introduction |
| Patient Information | **5a** | De-identified patient specific information. | p.2, section 2.1 |
| Patient Information | **5b** | Primary concerns and symptoms of the patient. | p.2, section 2.1 |
| Patient Information | **5c** | Medical, family, and psychosocial history including relevant genetic information. | p.2, section 2.1 |
| Patient Information | **5d** | Relevant past interventions and their outcomes. | p.2, section 2.1; Table 1 |
| Clinical Findings | **6** | Describe the relevant physical examination findings. | p.2, section 2.1 |
| Timeline | **7** | Depict important dates and times in this case in a table or figure. | Table 1 |
| Diagnostic Assessment | **8a** | Diagnostic methods, such as physical examination, laboratory testing, imaging, and surveys. | p.2-3, section 2.2 |
| Diagnostic Assessment | **8b** | Diagnostic challenges, such as financial, language, or cultural challenges. | p.3, section 2.2 |
| Diagnostic Assessment | **8c** | Diagnostic reasoning including other diagnoses considered. | p.3, section 2.2; Discussion |
| Diagnostic Assessment | **8d** | Prognostic characteristics, such as staging, where applicable. | p.3, section 2.2; Discussion |
| Therapeutic Intervention | **9a** | Types of therapeutic intervention, such as pharmacologic, surgical, preventive, or self-care. | p.3, section 2.3 |
| Therapeutic Intervention | **9b** | Administration of therapeutic intervention, such as dosage, strength, and duration. | p.3, section 2.3 |
| Therapeutic Intervention | **9c** | Changes in therapeutic intervention with rationale. | p.3, section 2.3 |
| Follow-up and Outcomes | **10a** | Clinician- and patient-assessed outcomes when appropriate. | p.3, section 2.3; p.5, section 5 |
| Follow-up and Outcomes | **10b** | Important follow-up diagnostic and other test results. | p.3, section 2.3; Table 1 |
| Follow-up and Outcomes | **10c** | Intervention adherence and tolerability, and how these were assessed. | p.3, section 2.3 |
| Follow-up and Outcomes | **10d** | Adverse and unanticipated events. | p.3, section 2.3 |
| Discussion | **11a** | Strengths and limitations of the approach to this case. | p.4-5, Discussion |
| Discussion | **11b** | Discussion of the relevant medical literature. | p.3-5, Discussion |
| Discussion | **11c** | The rationale for your conclusions. | p.4-5, Discussion |
| Discussion | **11d** | The primary take-away lessons from this case report. | p.5, Conclusion |
| Patient Perspective | **12** | When appropriate, the patient should share his or her perspective on the treatment received. | p.5, section 5 |
| Informed Consent | **13** | Did the patient give informed consent? Please provide if requested. | p.5, Ethics statement |
